# Radiological imaging characteristics of intramammary hematological malignancies: results from a german multicenter study

**DOI:** 10.1038/s41598-017-07409-z

**Published:** 2017-08-07

**Authors:** Susanne Wienbeck, Hans Jonas Meyer, Johannes Uhlig, Aimee Herzog, Sogand Nemat, Andrea Teifke, Walter Heindel, Fritz Schäfer, Sonja Kinner, Alexey Surov

**Affiliations:** 1 University Medical Center Göttingen, Institute for Diagnostic and Interventional Radiology, Robert-Koch-Str. 40, 37075 Göttingen, Germany; 2 University Hospital Halle, Department of Radiology, Ernst-Grube-Str. 40, 06120 Halle, Germany; 3 University of Jena, Institute for Diagnostic and Interventional Radiology, Erlanger Allee 101, 07747 Jena, Germany; 4 University of Saarland, Institute for Diagnostic and Interventional Radiology, Kirrberger Str. 100, 66424 Homburg, Germany; 5 University of Mainz, Department of Diagnostic and Interventional Radiology, Langenbeckstr. 1, 55131 Mainz, Germany; 6 University of Muenster, Institute for Clinical Radiology, Albert-Schweitzer-Str. 33, 48149 Muenster, Germany; 7 University of Kiel, Institute for Radiology and Neuroradiology, Arnold-Heller-Str. 3, 24105 Kiel, Germany; 8 University of Essen, Institute for Diagnostic and Interventional Radiology and Neuroradiology, Hufelandstr. 55, 45147 Essen, Germany; 9 University of Leipzig, Department of Diagnostic and Interventional Radiology, Liebigstr. 20, 04103 Leipzig, Germany

## Abstract

To assess radiological procedures and imaging characteristics in patients with intramammary hematological malignancies (IHM). Radiological imaging studies of histopathological proven IHM cases from ten German University affiliated breast imaging centers from 1997–2012 were retrospectively evaluated. Imaging modalities included ultrasound (US), mammography and magnetic resonance imaging (MRI). Two radiologists blinded to the histopathological diagnoses independently assessed all imaging studies. Imaging studies of 101 patients with 204 intramammary lesions were included. Most patients were women (95%) with a median age of 64 years. IHM were classified as Non Hodgkin lymphoma (77.2%), plasmacytoma (11.9%), leukemia (9.9%), and Hodgkin lymphoma (1%). The mean lesion size was 15.8 ± 10.1 mm. Most IHM presented in mammography as lesions with comparable density to the surrounding tissue, and a round or irregular shape with indistinct margins. On US, most lesions were of irregular shape with complex echo pattern and indistinct margins. MRI shows lesions with irregular or spiculated margins and miscellaneous enhancement patterns. Using US or MRI, IHM were more frequently classified as BI-RADS 4 or 5 than using mammography (96.2% and 89.3% versus 75.3%). IHM can present with miscellaneous radiological patterns. Sensitivity for detection of IHM lesions was higher in US and MRI than in mammography.

## Introduction

Intramammary hematological malignancies (IHM) have been described as a inhomogeneous group of breast malignancies, including various subtypes of malignant lymphomas, plasmacytomas and leukemias^1–3^. Overall, breast involvement in malignant hematological diseases is an uncommon manifestation and accounts for approximately 0.04–0.5% of all malignant breast cancers^4, 5^. Of all extranodal malignant lymphomas, approximately 0.85–2.2% manifest as primary breast lymphomas^6–8^. Secondary breast involvement in a patient, who has a history of systemic malignant lymphoma, is more common^9–11^.

Breast involvement by lymphoma can present as a primary breast tumor, or as an extranodal manifestation in systemic disease^4–8^.

Diffuse large B-cell lymphoma is the most common histologic IHM subtype, followed by extranodal marginal zone lymphoma and follicular lymphoma^3, 5–7, 12, 13^. Other types of breast lymphoma include mucosa associated lymphoma^3, 6, 12^, Burkitt lymphoma^3, 6^, plasmacytoma^1, 6^ and T-cell lymphoma^12, 14^. In addition, there were reports of Hodgkin’s disease manifestation of the breast^15, 16^. Breast plasmacytoma has been reported to occur in 1.5% of all incident plasmocytoma cases, whereas it accounts for only 0.2% of all malignant breast cancers^1^. Intramammary relapse in leukemia varied from 1.1–6.5% in myeloid leukemia^17, 18^. The exact prevalence of breast leukemia is difficult to determine^14^. As reported previously, at the moment, there is a paucity of studies focusing on radiological characteristics of different IHM. Various IHM imaging characteristics have been described in the literature, complicating radiological assessment of a crucial breast disorder^5, 9, 19, 20^. Therefore, the purpose of our study was to assess radiological procedures and imaging characteristics of IHM in a large multicenter sample.

## Methods

### Data acquisition and patients

This retrospective study was initiated by the department of radiology of the Martin-Luther-university Halle-Wittenberg and has been approved by the institutional ethics committee. All methods were performed in accordance with the relevant guidelines and regulations. Written informed consent from the study patients was not required.

Intramammary hematological malignancies diagnosed at 10 University affiliated breast centers from 1997 to 2012 were retrospectively assessed. Only histologically proven cases of IHM diagnosed by breast biopsy were included. Breast lesions suspicious for IHM without histopathological examination were excluded. Hematological malignancies with pectoral or axillary lymphadenopathy without breast affection, osseous or soft tissue malignant hematological tumors with invasion into the breast were also excluded from the study.

### Imaging

All provided breast imaging studies were accumulated in digital format at the Department of Radiology of the University Hospital Halle and were analyzed by two radiologists (S.W. and A.H.) with 7 and 4 years’ experience in breast imaging in consensus reading. They were blinded to the patients’ information and unaware of histopathological diagnosis. All lesions were classified according to the Breast Imaging Reporting and Data Systems (BI-RADS) 5^th^ edition categories^21, 22^.

### Mammography

Mammography was available in 73 patients. It was obtained on different dedicated digital or analog mammographic equipment’s. In every case, a standard mediolateral oblique and craniocaudal view was made. The ACR breast density types of mammographic images was classified according to the BI-RADS^®^ lexicon^21, 22^.

### Ultrasound

Sonographic images in axial and transverse planes were available for 53 patients. Different linear-array transducers at a center frequency of 8–13 Megahertz (MHz) were used.

### Magnetic resonance imaging

Magnetic resonance imaging (MRI) was performed in 28 patients utilizing different 1.5 Tesla scanners. Data of kinetic analysis of contrast enhancement, without diffusion-weighted imaging was available for 24 patients. Time-signal intensity curves were drawn using operator defined region of interest (ROI). The ROI was smaller than the lesion size. The initial signal increase (Initial SI) from the pre-contrast value (SI_p_) to the maximum peak within the first 3 min after the administration of contrast medium (SI_1–3 min_) was calculated as reported previously^23^:

$${\rm{I}}{\rm{n}}{\rm{i}}{\rm{t}}{\rm{i}}{\rm{a}}{\rm{l}}\,{\rm{S}}{\rm{I}}({\rm{ \% }})=({{\rm{S}}{\rm{I}}}_{1{\textstyle \text{-}}3min}-{{\rm{S}}{\rm{I}}}_{{\rm{p}}})/{{\rm{S}}{\rm{I}}}_{{\rm{p}}}\times 100{\rm{ \% }}$$

The post-initial behavior of the signal curve (Post-initial SI) from the maximum peak (SI_peak_) to the end of the examination (SI_end_) was also analyzed:

$$\mathrm{Post} \mbox{-} \mathrm{initial}\,{\rm{SI}}( \% )=({{\rm{SI}}}_{{\rm{peak}}}-{{\rm{SI}}}_{{\rm{end}}})/{{\rm{SI}}}_{{\rm{peak}}}\times 100 \% $$

### Statistical analysis

Continuous variables were expressed as mean ± standard deviation (SD), and categorical variables as percentages. Patterns, lesions size and the number of the different IHM were analyzed by the non-parametric chi-square test. All statistical analyses were performed using the SPSS statistical software package (SPSS 17.0, SPSS Inc., Chicago IL, USA). The significance level was set as alpha = 0.05. All reported p-values are two-sided.

## Results

### Primary diagnosis and lesions

Overall, imaging findings of 101 patients with different IHM were included. There were 96 women (95%) and 5 men (5%) with a mean age of 61.8 ± 14.4 years, median age, 64 years, range, 22–84 years. In most cases (n = 78, 77.2%), breast involvement in non-Hodgkin-lymphoma (NHL) was diagnosed (Table 1). Other hematological malignancies were rarely diagnosed.

**Table 1:** Identified intramammary hematological malignancies (IHM).

Diagnosis	n (patients)	%
Non-Hodgkin-lymphoma (NHL)	78	77.2
Plasmacytoma	12	11.9
Leukemia	10	9.9
Hodgkin lymphoma	1	1.0
**Total**	101	100

Of the 78 cases with NHL, different B-cell lymphomas were identified in 75 patients (96.2%) and T-cell lymphoma in 3 patients (3.8%). Most frequently, diffuse large B-cell lymphoma was diagnosed (35 patients, 47.9%) (Table 2).

**Table 2:** Non-Hodgkin-lymphoma (NHL) subtypes affected the breast.

NHL	n (patients)	%
Diffuse large B-cell lymphoma	37	47.4
Follicular lymphoma	5	6.4
Burkitt lymphoma	4	5.1
Marginal zone lymphoma	3	3.8
Centroblastic lymphoma	2	2.6
Mucosa associated lymphoma	1	1.3
Non specified lymphoma	23	29.5
T-cell lymphoma	3	3.8
Total	78	100

The right breast was involved in 46 patients (45%), the left breast in 37 (37%), and bilateral involvement of the breast was evident in 18 patients (18%). A total of 204 lesions were identified in the 101 patients included in this study (average of 2.02 lesions per patient). In 54 patients (53%), the lesions were solitary, 47 patients (47%) presented with multiple IHM lesions. Although no statistically significant (p > 0.05), the number of lesions per patient varied across different IHM: 1.9 in NHL, 1.5 in leukemia, and 2.9 in plasmacytoma.

### Clinical presentation

Intramammary hematological malignancies presented with skin thickening in 9 patients (12.3%), axillary lymphadenopathy in 12 (16.4%), and nipple retraction in 7 patients (9.5%).

## Radiological Features

### Mammographic findings

Mammography was obtained in 73 patients, comprising 131 lesions. Most of them show a breast density type b (52.1%) or breast density type c (37%), as shown in Table 3.

**Table 3:** Mammographic findings of intramamamary hematological malignancies (IHM).

Mammographic patterns	Patients (n = 73)	(%)
Intramammary masses	47	64.4
Architectural distortion	15	20.5
Combined pattern	11	15.0
**Breast density category (5** ^**th**^ **BI-RADS** ^**®**^ **)**	**Patients (n** = **73)**	**(%)**
a	5	6.8
b	38	52.1
c	27	37.0
d	3	4.1
**BI-RADS** ^**®**^ **category**	**Patients (n** = **73)**	**(%)**
0	4	5.5
2	6	8.2
3	8	11.0
4	38	52.0
5	17	23.3

Three patterns of IHM were identified on mammography: intramammary masses (47 patients, 64.4%), architectural distortions (15 patients, 20.5%), and a combined pattern, i.e. architectural distortion and breast masses (11 patients, 15%). Micro- or macrocalcifications were not identified on mammographic imaging of IHM.

Although not significant (p > 0.05), the frequency of the mammographic patterns was different in several IHMs, which are summarized in Table 4.

**Table 4:** Mammographic patterns in different intramammary hematological malignancies (IHM).

Mammographic findings	Leukemia n** (%)	NHL n** (%)	Plasmacytoma n** (%)
Intramammary masses	3 (42.9)	37 (62.7)	7 (100)
Architectural distortion	3 (42.9)	12 (20.3)	—
Combined pattern	1 (14.2)	10 (17.0)	—
**Total**	**7 (100)**	**59 (100)**	**7 (100)**

Notes: *There were no significant differences in patterns between the IHM groups; P > 0.05.

** n = number of patients.

**Figure 1:**
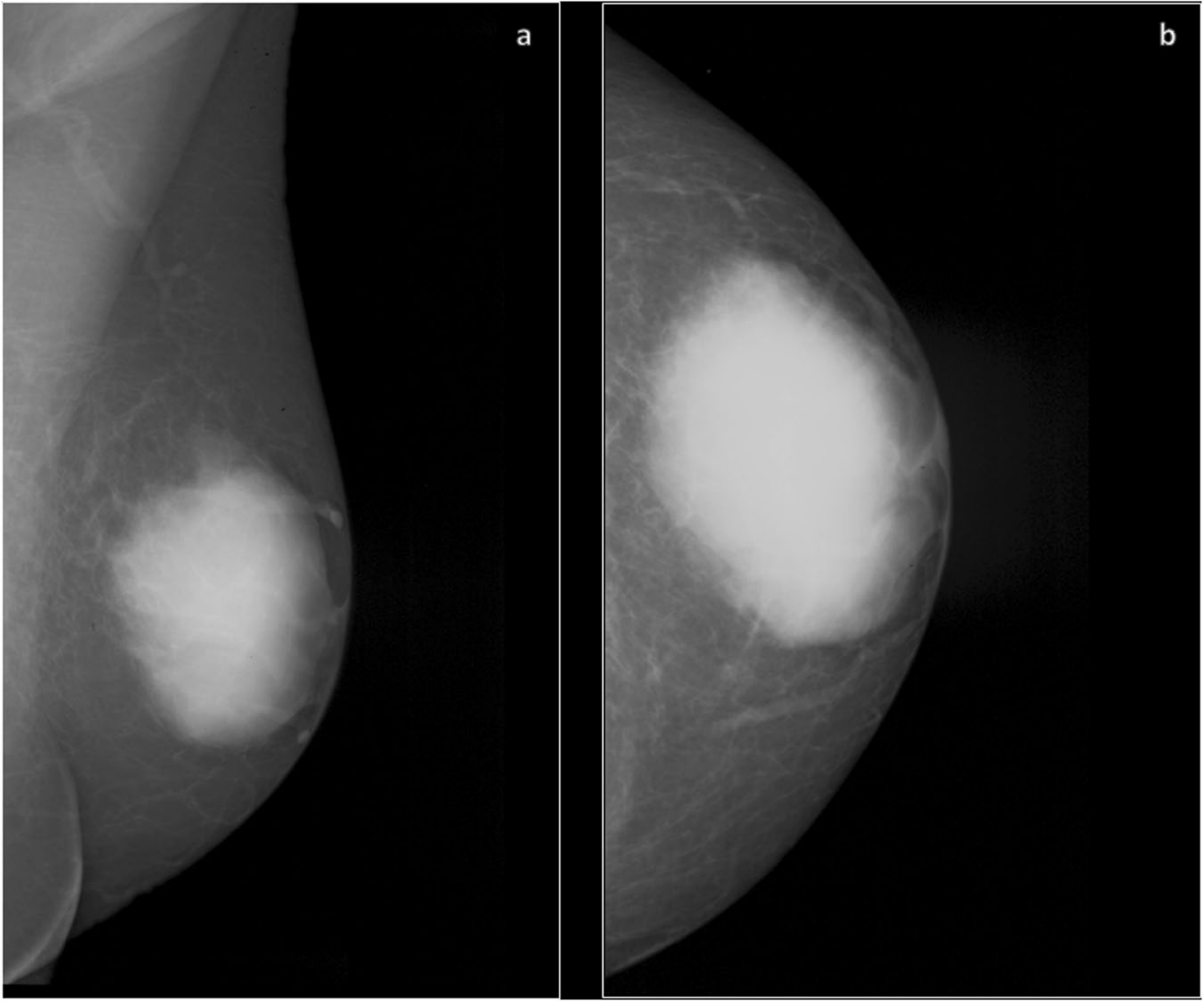
Mammographic findings of a breast plasmacytoma in a 54-year old man. Mammography of the left breast in mediolateral oblique (**a**) and craniocaudal (**b**) views showing a high-density mass with indistinct margins in the central part of the breast.

**Figure 2:**
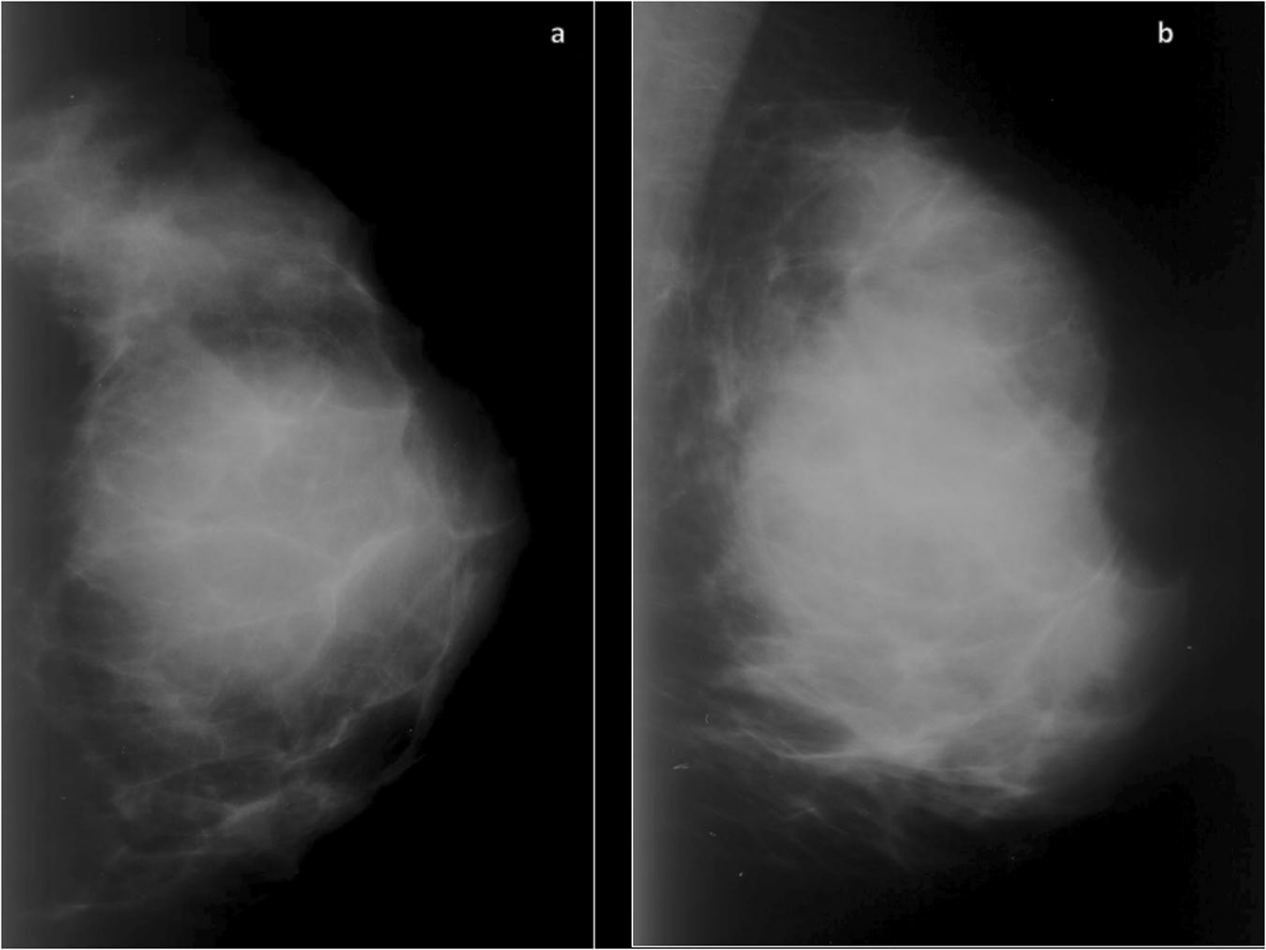
Mammographic findings of a diffuse large B-cell lymphoma in a 48-year old woman. The craniocaudal (**a**) and mediolateral oblique (**b**) mammogram of the left breast shows a high-density mass obscured by the surrounding breast parenchyma with indistinct margins infiltrating the whole breast.

Solitary or multiple intramammary masses were the most frequent mammographic finding in the patients (Fig. 1). Overall, 131 masses were identified. They were most commonly round or irregular in shape (101/77.1%) with indistinct margins, as demonstrated in Table 5. Furthermore, most lesions had an equal density (90/68.7%) to the surrounding tissue (Fig. 2). The mean size of the identified lesions was 15.8 ± 10.1 mm (median size 15 mm, range 5–60 mm). There were no significant differences in size between several IHMs (p > 0.05).

**Table 5:** Mammographic features of IHM masses (n = 131 lesions).

Mammographic findings	Lesions (n = 131)	(%)
**Shape**		
oval	30	22.9
round	57	43.5
irregular	44	33.6
**Margin**		
circumscribed	23	17.6
obscured	19	14.5
microlobulated	3	2.3
indistinct	78	59.5
spiculated	8	6.1
**Density**		
equal	90	68.7
high	41	31.3

Mammographic imaging studies were classified as BI-RADS 0 in 4 patients (5.5%), BI-RADS 2 in 6 patients (8.2%), BI-RADS 3 in 8 patients (11%), BI-RADS 4 in 38 patients (52%), and BI-RADS 5 in 17 patients (23.3%), (Table 3) by consensus, blinded reading.

### Ultrasound findings

**Figure 3:**
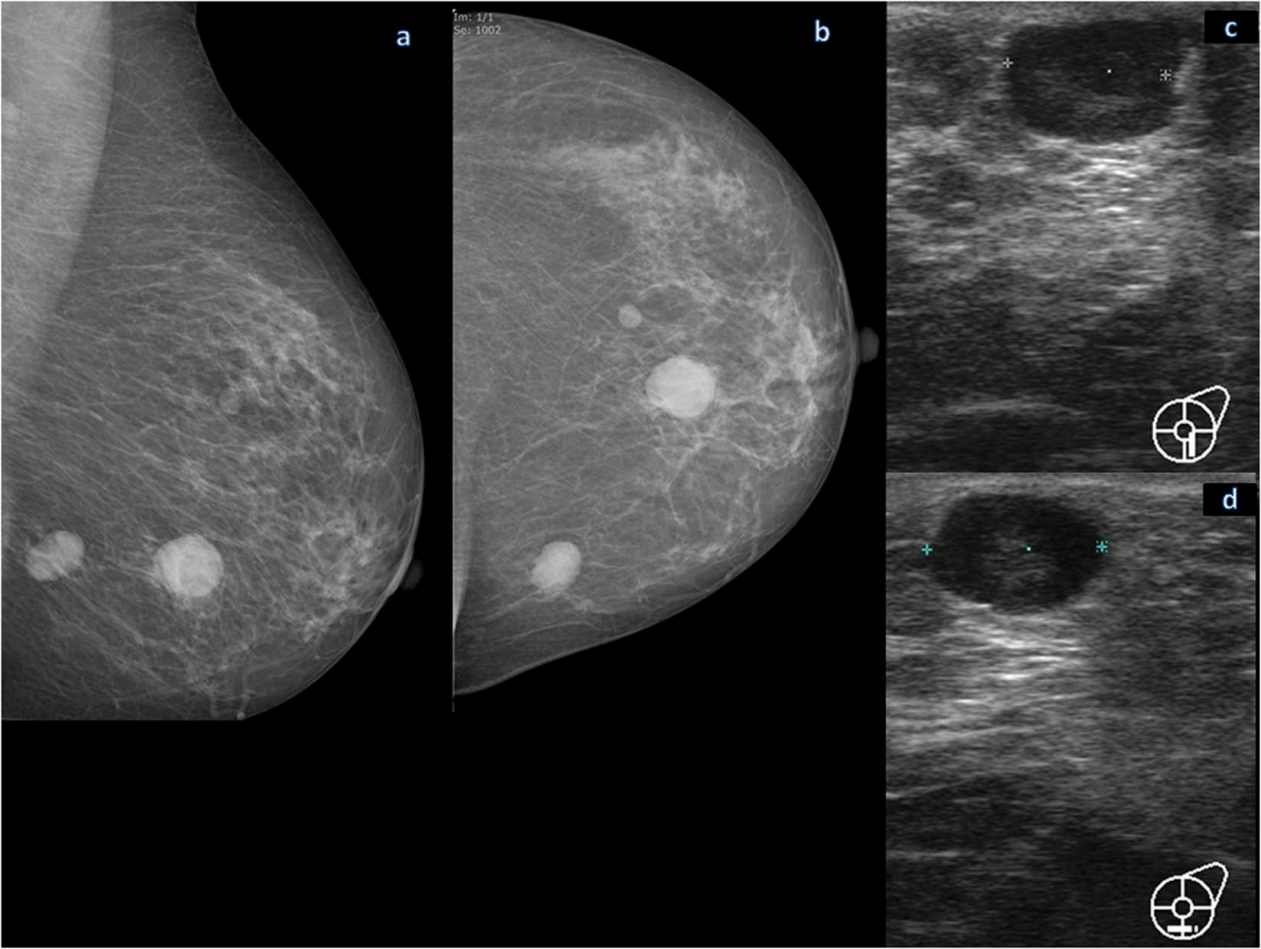
Mammographic and ultrasound findings of a leukemic relapse (acute lymphatic leukemia) affecting the left breast in a 34-year old woman. Mediolateral oblique (**a**) and craniocaudal (**b**) mammogram of the left breast shows three oval or round high-density masses with circumscribed margins. No associated skin thickening or ipsilateral axillary lymphadenopathy is present. The ultrasound findings in vertical (**c**) and horizontal (**d**) imaging planes demonstrated a circumscribed, oval mass with a complex cystic and solid echo pattern with posterior acoustic enhancement, located in the 6 o’clock position of the left breast.

**Figure 4:**
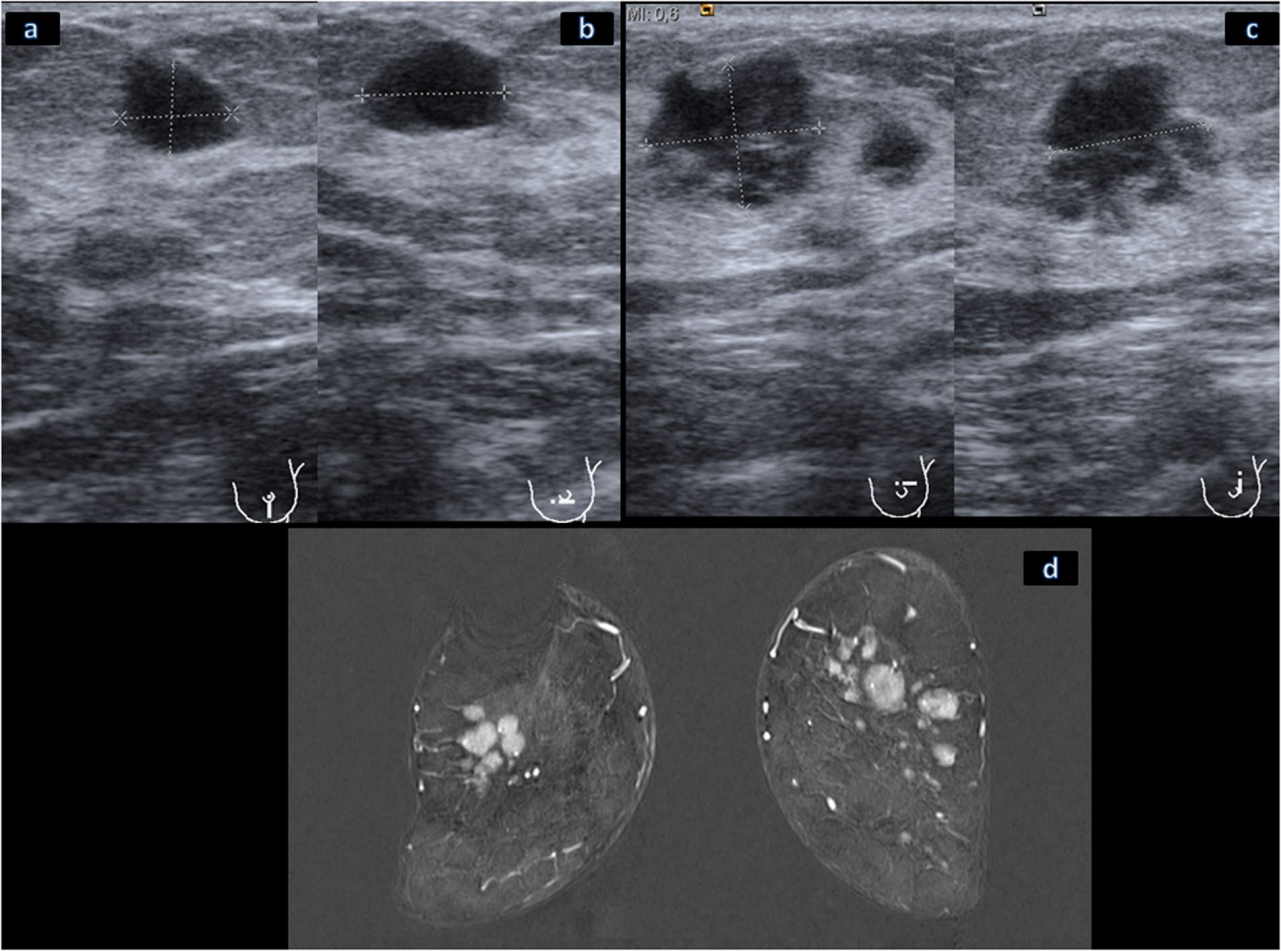
Ultrasound imaging findings in a 64-year old woman with breast T-cell lymphoma. The horizontal (**a**) and vertical (**b**) imaging planes demonstrated an oval, hypoechogenic mass with indistinct margins and without posterior acoustic features, located in the 6 o’clock position of the left breast. Another lesion in the upper outer quadrant of the left breast demonstrated in the vertical and horizontal imaging planes an irregular, complex cystic and solid mass with indistinct margins and with combined posterior acoustic pattern (**c**). The MRI imaging in coronal view (**d**) documented in the subtraction images multiple round and oval intramammary masses of both breasts with irregular margins and a homogeneous intensive enhancement.

Ultrasound images were obtained in 53 patients with 95 lesions. The identified US features of IHM are shown in Table 6. The majority of lesions were irregular in shape (55 lesions, 57.9%) with complex echo pattern (56 lesions, 58.9%) and indistinct margins (68 lesions, 71.6%), (Figs 3 and 4). Furthermore, most lesions had a non-parallel orientation (55 lesions, 57.9%) and different posterior acoustic features. Most US findings were classified as BI-RADS 5 (38 lesions, 71.7%).

**Table 6:** Ultrasound findings in IHM (n = 95 lesions).

Ultrasound findings	Lesions (n = 95)	(%)
**Shape**		
oval	31	32.6
round	9	9.5
irregular	55	57.9
**Orientation**		
parallel	40	42.1
non parallel	55	57.9
**Margin**		
circumscribed	13	13.7
microlobulated	3	3.1
indistinct	68	71.6
spiculated	11	11.6
**Echo pattern**		
complex cystic and solid	56	58.9
hypoechoic	36	37.9
isoechoic	3	3.2
**Posterior features**		
absent	43	45.3
enhancement	8	8.4
shadowing	28	29.5
combined pattern	16	16.8
**BI-RADS** ^**®**^ **category (n = 53 patients)**		
2	1	1.9
3	1	1.9
4	13	24.5
5	38	71.7

### MRI findings

MRI features of IHM are summarized in Table 7. Overall, 28 patients with 36 intramammary findings were investigated by MRI. Lesions presented either as masses (86.1%) or as non-mass enhancing lesions (13.9%). Non-mass enhancement manifested as diffuse (n = 2), segmental (n = 1) or localized in multiple regions (n = 2). Most IHM masses presented with irregular shape, irregular or spiculated margins, and various enhancement patterns (Fig. 4). All lesions showed an intermediate signal on T2 weighted images compared to breast parenchyma. Table 4 presents IHM MRI findings.

**Table 7:** MRI findings in IHM.

MRI findings	n (%)
**Mass**	31 (86.1)
**Non-mass enhancement**	5 (13.9)
**Intramammary masses**	
**Features**	**n (%) of masses**
**Shape**	
oval	7 (22.6)
round	4 (12.9)
irregular	20 (64.5)
**Margin**	
smooth	5 (16.1)
irregular	23 (74.2)
spiculated	3 (9.7)
**Internal enhancement characteristics**	
rim enhancement	3 (9.7)
heterogeneous	12 (38.7)
homogeneous	14 (45.2)
dark internal septations	2 (6.4)
**Kinetic curve assessment (n** = **24)**	
**Initial phase**	
fast	24 (100)
**Delayed phase**	
plateau	20 (83.3)
washout	4 (16.7)
**Signal intensity on T2 weighted images**	
intermediate	24 (100)
**BI-RADS** ^**®**^ **category (28 patients)**	**n (%)**
2	3 (10.7)
4	7 (25.0)
5	18 (64.3)

Kinetic analysis of contrast enhancement was performed for 24 masses. It showed a rapid initial signal increase (Initial SI) over 100% in comparison with pre-contrast signal intensity in all lesions. In the delayed phase, a plateau was seen in 20 cases (83.3%). In 4 cases (16.7%), a washout phenomenon was noted.

On MRI, the IHM lesions were classified as BI-RADS 2 in 3 patients (10.7%), BI-RADS 4 in 7 patients (25%), and BI-RADS 5 in 18 patients (64.3%).

## Discussion

### Primary diagnosis and patients

The present study evaluates radiological procedures and imaging characteristics of IHM based on a large sample of 101 patients with 204 lesions acquired in 10 University affiliated breast imaging centers. Previous reports regarding IHM reported on 11 to 36 patients^5, 14, 16, 20^. Furthermore, the studies primarily analyzed primary or secondary lymphomas of the breast, whereas other IHM subtypes, such as breast plasmacytoma or leukemia were not reported.

In our study, IHM predominantly manifests women, which is supported by the literature^24, 25^. These results were concordant with our data.

Patients median age at IHM diagnosis was 64, ranging from 22-84 years, consistent with earlier reported median age and age ranges^4, 8, 11, 26–28^.

Most case series on smaller patient cohorts documented a propensity for breast lymphoma manifestation in the right breast^4, 5, 26, 29, 30^. However, in the present large scale study, no side predominance of breast lymphoma localization was seen. Bilateral breast involvement was evident in 18% of cases.

### Mammographic findings

In the present study, intramammary masses were the most common mammography pattern of IHM. Most of the masses were round or irregular in shape with indistinct margins. There was no significant difference in shape and margin characteristics of lesions between the different types of the IHM. Furthermore, other patterns of IHM were identified: In 21% of cases, breast lymphoma presented on mammography as architectural distortion (focal or global asymmetry), especially in cases with NHL and leukemia. This pattern was not detected in intramammary plasmacytomas. In addition, a combined pattern of architectural distortion and masses was found in 15% of cases. This manifestation has not been reported previously. The frequency of mammographic patterns varied across several IHMs, although not statistically significant.

In concordance to our findings, mammographic findings of breast lymphomas are heterogeneous and lack a pathognomonic presentation, as described by a smaller case series^5^. In general, breast masses were the most common radiologic features observed in IHM^5, 20, 31^.

In agreement with our results, earlier studies described that on mammography most masses presented lobular or irregular with indistinct margins and variable density^5, 20, 29, 32^. Typically, no masses had calcifications or spiculated margins^5, 9, 16, 20, 29, 30, 32^. However, there are reports on IHMs manifesting as oval solitary or multiple masses with high-density and well-circumscribed margins, thereby mimicking benign lesions^20, 26, 29, 33^.

Surprisingly, axillar lymphadenopathy is not a prominent feature of most reported cases, but was shown in 16.4% of our patients^5, 14, 26, 34–36^.

In our study collective the mammograms of 14 patients (19.2%) were categorized as BI-RADS category 2 or 3. These results are in concordance with previous reports^14^. This may be related to the fact that breast lesions in several malignant hematological diseases can mimic benign disorders on mammography. Overall, published case series described comparably small cohorts of 10 to 96 lesions^5, 9, 14, 16, 19, 20, 23, 26, 29^. Therefore, our study with 131 mammographic findings in 73 patients with IHM is the largest to date.

### Ultrasound findings

In our series based on 95 lesions, 55 (57.9%) were irregular in shape with indistinct margins (71.6%). Most lesions had complex echo patterns with a non-parallel orientation and a broad spectrum of posterior acoustic phenomenon.

Contrasting our findings, the literature describes uniform sonographic appearances with hypoechoic round or oval masses, increasing the risk for false negative diagnoses^14, 16, 20^. However, these studies were based on smaller cases series of 5 to 24 patients.

In agreement with previous reports^14^, ultrasound had a higher proportion of BI-RADS category 5 lesions (71.7%) than mammography (23.3%). This finding suggests that patients with hematological disorders and intramammary lesions should be clarified by both modalities: mammography and ultrasound.

### MRI findings

In the literature, MRI findings of IHM are mainly presented as case reports, with only three studies describing MRI findings in 7 to 23 lesions^14, 20, 37–40^.

In our study, overall 36 intramammary findings were analyzed by MRI. IHM mainly presented as an oval breast masses (31/36, 86.1%), which was consistent with the results by Rizzo *et al*.^37^ and Liu *et al*.^38^. On T2 weighted images, all investigated lesions appeared intermediate compared to breast parenchyma, in line with the literature^14, 37^.

Furthermore, most of the lesions showed malignant features, such as an irregular shape with irregular margins, complicating the discrimination from other malignant breast tumors.

After intravenous administration of contrast medium, IHM internal enhancement was mostly homogeneous or slightly heterogeneous. As reported previously, rim enhancement and dark internal septation were rarely seen^14, 37, 38, 41^, which is similar to the enhancement pattern of lymphomas in other sites. Overall, MRI findings of IHM differ from those in frequent breast carcinomas, which usually show marked heterogeneous enhancement^42, 43^.

In most reports, only qualitative analysis of the enhancement kinetic was performed^14, 37^. Studies reported comparable kinetic curve patterns with a rapid enhancement and a plateau in the delayed phase^14, 37, 38^. However, these findings were obtained by 1 Tesla MRI scanners^14, 37^.

In our study, kinetic analysis of contrast enhancement was performed in 24 lesions. A rapid initial signal increase over 100% in comparison with pre-contrast signal intensity was seen in all cases. The delayed phase showed a plateau phenomenon in most cases (83.3%). These results were in agreement with earlier studies^37, 38^. However, this finding is generically expected for all malignant lesions, regardless of histological type, and is therefore not pathognomonic for breast lymphoma^44, 45^.

In our study, MRI yielded a higher proportion of BI-RADS category 5 lesions (64.3%) than mammography (23.3%). Presumably, MRI sensitivity for the detection of multifocal or multicentric tumors might be superior to other modalities. Thus, in the case of suspected or confirmed breast lymphoma, MRI could be recommended for a more accurate and comprehensive assessment.

### Strengths

Our study had several strengths. We present the largest report on IHM with 101 patients and a total of 204 lesions. Moreover, blinded two-reader assessment of various modalities ensured a comprehensive characterization of radiological IHM features. Finally, the multi-center setting of our study yields information on IHM imaging findings generalizable to the population, minimizing local influences.

### Limitations

There are several limitations to our study. First, the retrospective setting limits analyses. Second, not all imaging modalities of ultrasound, mammography, and MRI were available for every patient. However, this algorithm reflects the typical workflow in clinical practice, where breast lesions are subjected to percutaneous biopsy without complete imaging work-up on all imaging modalities.

## Conclusion

In conclusion, our study demonstrated imaging findings of IHM based on the largest sample to date. IHM manifested most frequently as a lobular or irregular mass with indistinct margins at mammography and as hypoechoic, irregular mass with indistinct margins and different posterior acoustic phenomena at ultrasound. On MRI, the identified lesions are variable in shape and show rapid contrast enhancement with post-initial plateau. Furthermore, the imaging features of breast lesions in IHM are more typically malignant in ultrasound and MRI than in mammography.
